# Calcitriol Administration Attenuates Acute Lung Injury by Improving Angiopoietin/Tie2 Dysregulation and Alveolar-Capillary Barrier Integrity in a Mouse Model of Ovariectomy Complicated With Sepsis

**DOI:** 10.1155/mi/4374164

**Published:** 2025-10-07

**Authors:** Chiu-Li Yeh, Shang-Ming Tseng, Mei-Pei Huang, Wei-Hsin Lin, Ting-Chun Kuo, Jin-Ming Wu, Kuen-Yuan Chen, Ming-Hsun Wu, Po-Jen Yang, Po-Chu Lee, Chien-Chia Chen, Chih-Yuan Lee, Sung-Ling Yeh, Ming-Tsan Lin

**Affiliations:** ^1^School of Nutrition and Health Sciences, College of Nutrition, Taipei Medical University, Taipei, Taiwan; ^2^Research Center of Geriatric Nutrition, College of Nutrition, Taipei Medical University, Taipei, Taiwan; ^3^Department of Traumatology, National Taiwan University Hospital and College of Medicine, National Taiwan University, Taipei, Taiwan; ^4^Department of Surgery, National Taiwan University Cancer Center, Taipei, Taiwan; ^5^Department of Surgery, National Taiwan University Hospital Jinshan Branch, New Taipei City, Taiwan; ^6^Department of Surgery, National Taiwan University Hospital and College of Medicine, National Taiwan University, Taipei, Taiwan

**Keywords:** Ang1/Ang2 ratio, cuproptosis, ferroptosis, redox status, survival rate, tight junction

## Abstract

Menopause is associated with excessive weight, increased systemic inflammation, and oxidative stress. Menopause with obesity may aggravate the severity of organ damage when complicated with sepsis. This study investigated the impacts of calcitriol on acute lung injury (ALI)-associated angiopoietin (Ang)/tyrosine kinase with immunoglobulin-like and epidermal growth factor-like domain 2 (Tie2) dysregulation, impairment of the barrier integrity, and ion disturbance in ovariectomized mice with sepsis. 6-month-old female C57BL/6 mice were divided into three groups: the OB group, which contained mice with a sham ovariectomy and high-fat diet (HFD); the OVSS group, which contained mice with an ovariectomy, an HFD, cecal ligation and puncture (CLP), and an intravenous saline injection after CLP; and the OVSD group, which contained mice with an ovariectomy, an HFD, CLP, and a calcitriol injection. The HFD was fed for 12 weeks. Mice in the respective groups were sacrificed on 24 or 72 h after CLP. Results showed that compared to the OB group, the OVSS group had higher oxidative stress, iron and copper overload, whereas the Ang1/Ang2 ratio and tight junction (TJ) protein levels were lower in the lungs. Calcitriol treatment in mice with ovariectomy and CLP enhanced Tie2 levels and the Ang1/Ang2 ratio, increased zona occludens (ZOs)-1, occludin, and claudin-5 levels. Elevated reduced glutathione (GSH) levels and glutathione peroxidase 4 (GPX4) activities, reduced iron and copper contents, and suppressed ferroptosis- and cuproptosis-associated components were found in the lungs. Also, higher survival rates and lower lung injury scores were noted in the OVSD groups. These findings suggest that calcitriol treatment mitigated the dysregulated Ang/Tie2 pathway, improved the integrity of the alveolar-capillary barrier, elicited a more balanced redox status, alleviated ferroptosis and cuproptosis in the lungs in an ovariectomy model complicated with sepsis.

## 1. Introduction

Menopause is a natural part of the aging process resulting from the loss of ovarian function and a decline in circulating estrogen concentrations. Lack of estrogen's actions predisposes individuals to obesity and various metabolic disorders [1]. A previous study reported that peri and postmenopause periods were associated with increased systemic inflammation and lipid peroxidation, and an altered antioxidant defense ability in women [2]. It was also estimated that 70% of perimenopausal women are overweight or obese in the United States [3]. Obesity is a disease with chronic low-grade inflammation, oxidative stress, and dysregulated immune responses [4]. Menopause with obesity is associated with increased morbidity that may aggravate the severity of organ damage in critical conditions [3].

Sepsis is a lethal syndrome that commonly occurs in intensive care units (ICUs). Insults triggered by bacteria and their toxins lead to immune dysregulation and multiple organ failure [5]. Among the injured organs, the lungs are the most frequently affected during sepsis [6]. One of the main mechanisms of sepsis-induced lung damage is microvascular leakage, resulting from endothelial dysfunction and impairment of the barrier integrity [7]. Angiopoietin (Ang)/tyrosine kinase with immunoglobulin-like and epidermal growth factor-like domain 2 (Tie2) pathway plays an essential role in regulating vascular stability and integrity. Disturbance of the Ang/Tie2 axis causes endothelial activation and vascular leakage that leads to sepsis [7, 8]. On the other hand, intense metabolic stress during sepsis may lead to disturbances of ions that are involved in sepsis-related organ damage. Ferroptosis and cuproptosis are forms of programmed cell death resulting from metal ion overload. Ferroptosis is characterized by an accumulation of iron ions and lipid peroxidation products [9]. Cuproptosis is copper-dependent cell death originating from the direct binding of copper to lipoylated enzymes of the tricarboxylic acid (TCA) cycle that leads to proteotoxic stress [10]. A previous study found that ferroptosis contributes to the development of acute lung injury (ALI), and inhibition of ferroptosis alleviates sepsis-induced ALI [11]. Also, a recent study showed that cuproptosis-related genes are significantly associated with the occurrence and progression of sepsis-induced ALI [12].

Vitamin D (VD) is a fat-soluble vitamin with anti-inflammatory and immune-regulatory properties [13]. Previous studies showed that interventions with the active form of VD, 1,25(OH)_2_ D_3_ (calcitriol), modulate the homeostasis of T cell subsets and renin angiotensin system-associated signal pathways, thereby mitigating sepsis- and poly(I:C)-induced ALI [14–17]. In this study, we investigated the impact of calcitriol on the ALI-associated dysregulation of Ang/Tie2 signal pathways and barrier dysfunction in obese menopausal mice complicated with sepsis. The effects of calcitriol on ferroptosis- and cuproptosis-associated ALI were also investigated. An estrogen deficiency in menopause was created by performing an operation of an ovariectomy, and then a high-fat diet (HFD) was provided to induce obesity. Cecal ligation and puncture (CLP) was used in this study to induce murine polymicrobial sepsis. This model has been extensively used to investigate sepsis-associated organ injury and the underlying mechanisms [18]. A recent study reported that calcitriol alleviates sepsis-induced liver ferroptosis [19]. Also, the VD receptor (VDR) was reported to be involved in inhibiting airway epithelial ferroptosis, thus improving ALI [20]. Although no study has investigated the role of VD in cuproptosis at present, ferroptosis and cuproptosis are interrelated in terms of occurrence, signal pathways, and cell metabolism [21]. We hypothesized that calcitriol administration would alleviate ALI by modulating the Ang/Tie2 axis, improving the alveolar-capillary barrier integrity, and mitigating ferroptosis and cuproptosis in obese ovariectomized mice complicated with sepsis. Components related to Ang/Tie2 signaling, tight junction (TJ) proteins, and markers of ferroptosis and cuproptosis in the lungs were analyzed in this study.

## 2. Materials and Methods

### 2.1. Animals

Female C57BL/6 mice were used for the experiments. All animals were obtained from the National Laboratory Animal Center (NLAC, Taipei, Taiwan) and were housed in a room at 22 ± 2°C under lighting control with a 12-h light–dark cycle. Standard rodent chow and tap water were fed ad libitum for 6 months (weighing ~ 25 g). Six months of age is considered middle-aged in mice [21]. The mice were moved to the Animal Laboratory Center at Taipei Medical University (TMU) and provided with a chow diet ad libitum for 1 week before beginning the study. Animal experiments and procedures were performed in accordance with national guidelines for the use and care of laboratory animals, and were approved by the Institutional Animal Care and Use Committee of TMU (LAC-2023-0207).

### 2.2. Experimental Design

After 1 week of acclimation, mice with comparable weights were randomly assigned to three experimental groups: an OB group containing mice with a sham ovariectomy operation which were fed a 45% HFD (*n* = 8); an OVSS group containing mice with an ovariectomy and HFD, after which sepsis was induced and saline was injected via a tail vein 1 h after sepsis (*n* = 20); and an OVSD group containing mice with an ovariectomy and HFD, after which sepsis was induced and calcitriol (410 ng/kg body weight (BW); Cacare injection, Nang Kuang Pharmaceutical, Taipei, Taiwan) was injected 1 h after sepsis (*n* = 20). The dosage of calcitriol used was referenced from a clinical study that was reported to be well-tolerated and had benefits in sepsis patients [22]. Dose conversion between humans and mice was calculated according to the practice guideline [23]. This dosage was proven to have immunomodulatory and anti-inflammatory effects in mice with critical conditions [14, 16]. A bilateral ovariectomy was performed at 6 months of age. Mice were anesthetized using 2.5% isoflurane. A single incision in the skin of the dorsal lumbar area was made on each side. Each ovary was clamped and removed by a single cut. To relieve pain, bupivacaine (0.25%) was dripped onto the incision site. Then, the skin was closed with continuous sutures. The same procedures were conducted for the sham operation, but without removing the ovaries. After the respective operations, mice in the experimental groups were provided with an HFD for 12 weeks. The HFD was supplied by a commercial company (Research Diets, New Brunswick, NJ, USA), and its composition is presented in Table 1. The fat content in the diet and duration to induce obesity were referenced from a previous rodent model with an ovariectomy fed an HFD [24]. Then, sepsis was induced by CLP in the OVSS and OVSD groups. The CLP operation followed previously described procedures [14]. Briefly, mice were initially anesthetized, and then the cecum was ligated at a 30% distance from the distal pole. A 23-gauge needle was used to puncture through the cecum, and a small amount of feces was smeared onto the abdomen. Bupivacaine was dripped onto the incision site before skin closure. After the CLP operation, sterile saline was subcutaneously injected for rehydration. During the recovery phase, all mice were freely allowed to have water and their former diets. At the end of 12 weeks, mice in the obese group were anesthetized and euthanized by cardiac puncture, while mice in the sepsis groups were sacrificed on 24 or 72 h after CLP (*n* = 10 for each time point). These time points were chosen because 24 h is considered the late phase in the model of CLP [25], and the inflammatory response and injury to remote organs may sustain till 72 h after CLP [26, 27]. Plasma samples were obtained after centrifugation of the blood. A portion of lung tissue was used for a histological examination, and the remaining parts were frozen in liquid nitrogen until being processed for further analysis. For survival studies, 48 mice in total (OVSS, *n* = 28; OVSD, *n* = 20) were used. Survival was noted every 12 h until 72 h after CLP.

### 2.3. Measurements of Biochemical Parameters in Plasma

Cytokines and chemokines were analyzed using a ProcartaPlex Multiplex Immunoassay Kit (Invitrogen EPXR360-26092-901, Carlsbad, CA, USA). Adhering to the manufacturer's instructions, samples (25 µL) were processed on a MAGPI platform Milliplex Map, and data were collected with Luminex xPonent software (MilliporeSigma, Ontario, Canada). Results for interleukin (IL)-22, C-X-C motif chemokine ligand 10 (CXCL10), monocyte chemoattractant protein (MCP)-1, and MCP-3 were analyzed using the accompanying Analyst software, ensuring a comprehensive examination of the samples.

### 2.4. Analysis of Ang/Tie2-Associated Proteins in the Lungs

In total, 30 mg of lung tissue was homogenized in 300 µL of tissue protein extraction reagent (T-PER; Thermo Fisher Scientific, Vilnius, Lithuania), along with a protease and phosphatase inhibitor (Thermo Fisher Scientific). Homogenates were pulse-sonicated on ice for 20–30 s, then centrifuged at 12,000 rpm and 4°C for 10 min. The supernatants were collected, and the protein concentration was measured using a Bradford Protein Assay Reagent Kit (Bio-Rad, Hercules, CA, USA). Ang1, Ang2, vascular endothelial growth factor (VEGF), and Tie2 were analyzed using enzyme-linked immunosorbent assay (ELISA) kits (Ang1, OKEH00011; Ang2, OKEH05460; VEGF, OKRC01327; Tie2, OKEH00011, AVIVA Systems Biology, San Diego, CA, USA). Samples were diluted 400-fold for Ang1, 1600-fold for Ang2 and Tie2, and 50-fold for VEGF. Diluted samples were applied to 96-well plates according to the commercial product protocol. Results were adjusted based on the protein levels of each sample.

### 2.5. TJ Protein Levels in Lung Tissues

Supernatants derived from tissue homogenates were obtained as described above. After diluting the supernatants by 400-fold for claudin-5 and 200-fold for zona occludens (ZOs)-1 and occludin, mouse TJ protein 1, occludin, and claudin ELISA kits (with respective cat. nos. of EM1410, EM7786, and EM1956, FineTest, Wuhan, Hubei, China) were used to analyze TJ proteins. All protocols followed the guidelines provided in the manuals of the commercial kits. Data are expressed as pg/mg protein, and protein concentrations were measured using a commercial kit (Bio-Rad).

### 2.6. Analysis of Lipid Peroxide Levels in Lung Homogenates

4-Hydroxynonenal (4-HNE) is the primary lipid peroxidation product derived from polyunsaturated fatty acids (PUFAs) [28]. After diluting the supernatants 10-fold, a Lipid Peroxidation 4-HNE Assay Kit (ab238538, Abcam, Biomedical Campus, Trumpington, Cambridge, UK) was used to measure 4-HNE levels. Following the manufacturer's protocol, results were read at an optical density (OD) of 450 nm. The final results were adjusted based on protein concentrations in the supernatants. Protein levels were measured with a Bradford Protein Assay Reagent Kit (Bio-Rad).

### 2.7. Transferrin Receptor (TFRC) and Iron Contents in Lung Tissues

A Mouse Transferrin Receptor ELISA Kit (ab243674, Abcam) was used to analyze TFRC levels in tissues. The procedure was described in our previous report [19]. To analyze ferrous iron (Fe^2+^) in samples, an Iron Assay Kit (ab83366, Abcam) was used. Tissues were homogenized with five times the volume of acidic buffer (pH2009;<2009;5.5). Supernatants were collected and diluted 200-fold with buffer, followed by adding 5 µL of iron buffer and incubation. An iron probe was added and incubated, and the absorbance was read at an OD of 593 nm. The procedures followed the instruction manual and previously described details [19]. Concentrations of TFRC and iron were determined by interpolating absorbance values on a standard curve. Protein levels in the supernatants were analyzed by a Bradford Protein Assay Reagent Kit (Bio-Rad).

### 2.8. Reduced Glutathione (GSH) Levels and Glutathione Peroxidase 4 (GPX4) Activities in Lung Tissues

Fifty milligrams of lung tissues were homogenized in 0.5 mL buffer, including 50 mM Tris-HCl (J.T. Baker, Center Valley, PA, USA), 5 mM EDTA (Nacalai Tesque, Kyoto, Japan), and 1 mM DTT (Sigma–Aldrich, St. Louis, MO, USA) at pH 7.5. Total (*t*) GSH and GSH disulfide (GSSG) concentrations in supernatants were measured with commercial kits (Cayman Chemical, Ann Arbor, MI, USA) as mentioned in our previous report [19]. The GSH content was calculated as the difference between tGSH and GSSG. To measure GPX4 activity, 30 mg of tissue samples was homogenized in 0.3 mL phosphate-buffered saline (PBS) and stored overnight at −20°C. Two freeze-thaw cycles were performed to break up cell membranes, and the mixture was centrifuged to obtain the supernatant. Supernatants were used to analyze GPX4 activity by an ELISA Kit (cat. no. OKEH08828, Aviva Systems Biology, San Diego, CA, USA). Detailed procedures were followed as described previously [19]. GPX4 activity is presented as the relative absorbance by the difference between assay results and a mean blank well at 450 nm. The GSH content and GPX4 activities were adjusted by protein levels in the supernatant. Protein levels were analyzed by a Bradford Protein Assay Reagent Kit (Bio-Rad).

### 2.9. Analysis of Copper Ions in Lung Tissues

In total, 50 mg of flash-frozen lung tissue was homogenized using a loose-fitting Dounce homogenizer in 500 μL of a buffer solution that contained 0.1 mol/L sodium phosphate (pH 7.5) and 0.25 mol/L D-sucrose. The supernatants were used to analyze the copper (Cu^2+^) level using a Copper Assay Kit (cat. no. MAK127, Sigma–Aldrich), following the manufacturer's protocol. Final data are presented as Cu^2+^ concentrations (µg), adjusted according to protein levels in the supernatant. The protein level was measured with a Bradford Protein Assay Reagent Kit (Bio-Rad).

### 2.10. Messenger (m) RNA Extraction and Analysis With a Real-Time Reverse-Transcription (RT) Quantitative Polymerase Chain Reaction (qPCR) *in Lung Tissues*

Tissues were homogenized, and total RNA was isolated using the Trizol reagent (Invitrogen). RNA pellets were dissolved in RNase-free water and stored at −80°C for further analysis. RNA concentrations were quantified by absorbance at 260 and 280 nm with a spectrophotometer. A RevertAid first-strand complementary (c) DNA synthesis kit (Thermo Fisher Scientific) was used to synthesize cDNA. cDNA was stored at −80°C until being used. RT was performed by subsequent incubation for 5 min at 65°C, 60 min at 42°C, and 5 min at 70°C. mRNA genes were amplified by a real-time RT-PCR using the 7300 Real-Time PCR System (Applied Biosystems, Foster City, CA, USA) with SYBR Green I as the detection format. Genes measured included ferroptosis pathway-associated (nuclear factor erythroid 2-related factor 2 (*Nrf2*), ferroptosis-suppressing protein 1 (*FSP1*), solute carrier family 7 member 11 (*SLC7A11*), acyl-CoA synthase long chain family member 4 (*ACSL4*), and prostaglandin-endoperoxide synthase 2 (*PTGS2*)) and cuproptosis-associated genes (ferredoxin1 (*FDX1*), ATPase copper transporting alpha (*ATP7A*), *SLC31A1*, lipoic acid synthase (*LIAS*), and dihydrolipoyl transacetylase (*DLAT*)). Primers were based on deposited sequences (GenBank database, NCBI) provided by Mission Biotech (Taipei, Taiwan). All primers are presented in Table 2. A total volume of 25 µL containing Maxima SYBR Green/ROX qPCR Master Mix (2x) (Thermo Fisher Scientific), 100 ng of cDNA, and 40 nM of each primer was used for amplification. The reaction was processed by one cycle of 2 min at 50°C and 10 min at 95°C, followed by 40 cycles of 15 s at 95°C and 1 min at 60°C, with a final dissociation curve (DC). Mouse glyceraldehyde 3-phosphate dehydrogenase (GAPDH) was used as an internal control. Relative mRNA expression levels were calculated by cycle threshold (CT) values and normalized by dividing the result of the OS group at 12 h after CLP.

### 2.11. Histopathology of Lung Tissues

Lung tissues were fixed in 10% neutral phosphate-buffered formalin. Histopathological evaluations were conducted by a veterinary pathologist who was blinded to the study. Hematoxylin and eosin (H&E) staining of tissue slides was used to examine the morphologies of the specimens. Digital images at 200× magnification were captured for each section. The severity of lung injury was judged according to previous reports [29, 30]. The score severity was graded by the following histological features: (1) thickness of alveolar walls was graded as 0–4 and (2) percentages of lesion were graded as 0–3 (0: lesion affecting <3% of the area, 1: lesion affecting 4%–33%, 2: lesion affecting 34%–66%, and 3: lesion affecting >67%). The overall lung injury score was calculated as the sum of the two histologic score grades.

### 2.12. Statistical Analysis

The investigators were blinded during the variable parameter assessment. All data are presented as the mean ± standard error of the mean (SEM). GraphPad Prism 5 statistical software (GraphPad Software, La Jolla, CA, USA) was used to analyze the data. Survival curves were compared using a log-rank test. Comparisons among the OB and the septic groups at each time point used a two-way ANOVA followed by the Bonferroni post hoc test. A *p*-value of <0.05 was considered statistically significant.

## 3. Results

### 3.1. Lung Weights of the Experimental Groups

**Figure 1:**
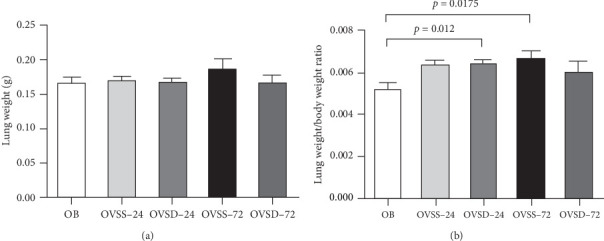
(A) Lung weights and (B) the ratio of lung weight/body weight among the experimental groups. OB, mice with a sham-ovariectomy operation and a high-fat diet (HFD) (*n* = 8); OVSS, mice with an ovariectomy, an HFD, and then cecal ligation and puncture (CLP) was performed, and saline was injected after CLP (*n* = 16), sacrificed on either 24 h (OVSS-24) or 72 h (OVSS-72); and OVSD, mice with an ovariectomy, an HFD, and CLP, and calcitriol was injected after CLP (*n* = 16), sacrificed on either 24 h (OVSD-24) or 72 h (OVSD-72). Values are expressed as the mean ± SEM. Comparisons among groups were analyzed by a two-way ANOVA followed by the Bonferroni post hoc test. There were no differences in lung weights among the groups (*p* > 0.05). The OB group had a lower lung weight/body weight ratio than the OVSD-24 (*p*=0.012) and OVSS-72 groups (*p*=0.0175).

There were no differences in lung weights among the experimental groups (Figure 1A). The lung weight/BW ratios were significantly higher in the OVSD-24 and OVSS-72 groups than in the OB group. No differences in lung weight/BW ratios were found between the OVSS and OVSD groups at either time point (Figure 1B).

### 3.2. Calcitriol Treatment Reduced Plasma Inflammatory Indicators in Ovariectomized Mice With Sepsis

**Figure 2:**
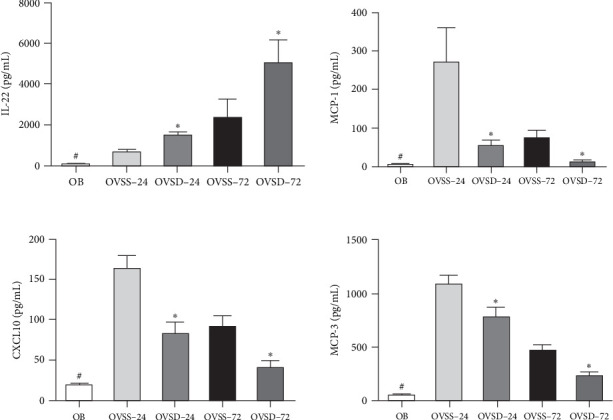
Plasma interleukin (IL)-22, C-X-C motif chemokine ligand 10 (CXCL10), monocyte chemoattractant protein (MCP)-1, and MCP-3 levels among the experimental groups. Calcitriol treatment reduced CXCL10, MCP-1, and MCP-3 levels in ovariectomized mice with sepsis. OB, mice with a sham-ovariectomy operation and a high-fat diet (HFD) (*n* = 8); OVSS, mice with an ovariectomy, an HFD, and then cecal ligation and puncture (CLP) was performed, and saline was injected after CLP (*n* = 16), sacrificed on either 24 h (OVSS-24) or 72 h (OVSS-72); and OVSD, mice with an ovariectomy, an HFD, and CLP, and calcitriol was injected after CLP (*n* = 16), sacrificed on either 24 h (OVSD-24) or 72 h (OVSD-72). Values are expressed as the mean ± SEM. Comparisons among groups were analyzed by a two-way ANOVA followed by the Bonferroni post hoc test. ^#^Significantly differs from the OVSS groups at 24 and 72 h. *⁣*^*∗*^Significantly differs from the OVSS groups at the same time point (*p* < 0.05).

The OVSS group had higher IL-22, CXCL10, MCP-1, and MCP-3 levels than those in the OB group. Compared to the OVSS group, the concentration of IL-22 was significantly higher, whereas CXCL10, MCP-1, and MCP-3 were lower in the OVSD groups on either 24 or 72 h after CLP (Figure 2).

### 3.3. Ang/Tie2 Pathway-Associated Protein Levels in Lung Tissues

**Figure 3:**
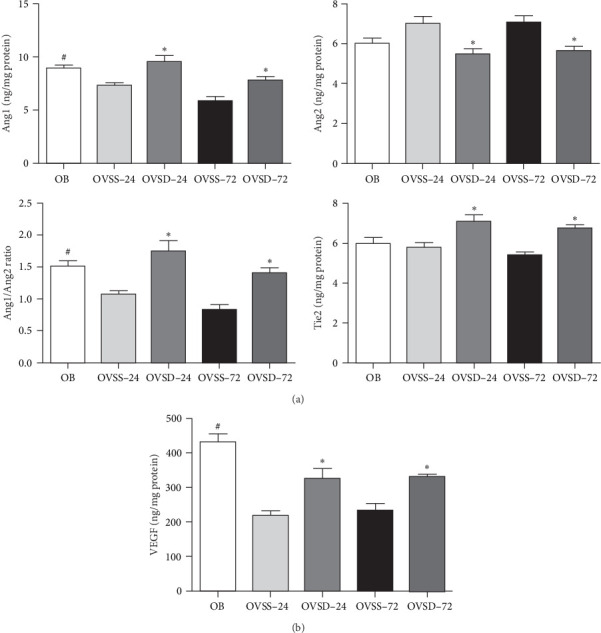
(A) Angiopoietin (ang)/tyrosine kinase with immunoglobulin-like and epidermal growth factor-like domain 2 (Tie2) pathway-associated proteins and (B) vascular endothelial growth factor (VEGF) levels in lung tissues. Calcitriol treatment showed higher Ang1, Tie2, and VEGF levels and an Ang1/Ang2 ratio in ovariectomized mice with sepsis. OB, mice with a sham-ovariectomy operation and a high-fat diet (HFD) (*n* = 8); OVSS, mice with an ovariectomy, an HFD, and then cecal ligation and puncture (CLP) was performed, and saline was injected after CLP (*n* = 16), sacrificed on either 24 h (OVSS-24) or 72 h (OVSS-72); and OVSD, mice with an ovariectomy, an HFD, and CLP, and calcitriol was injected after CLP (*n* = 16), sacrificed on either 24 h (OVSD-24) or 72 h (OVSD-72). Values are expressed as the mean ± SEM. Comparisons among groups were analyzed by a two-way ANOVA followed by the Bonferroni post hoc test. ^#^Significantly differs from the OVSS groups on 24 and 72 h. *⁣*^*∗*^Significantly differs from the OVSS group at the same time point (*p* < 0.05).

The OVSS group showed significantly lower Ang1 levels and Ang1/Ang2 ratio than those of the OB group. Compared to the OVSS group, the OVSD group had significantly higher Ang1 and Tie2, and lower Ang2 levels after CLP. A higher Ang1/Ang2 ratio was also noted in the OVSD groups than the OVSS groups at both time points after CLP (Figure 3A). The levels of VEGF in the OVSS groups were lower than those in the OB group. The OVSD groups had higher VEGF levels than those in the OVSS groups after CLP (Figure 3B).

### 3.4. Calcitriol Enhanced VDR Expression and TJ Protein Levels in Lung Tissues of the Ovariectomized Mice With Sepsis

**Figure 4:**
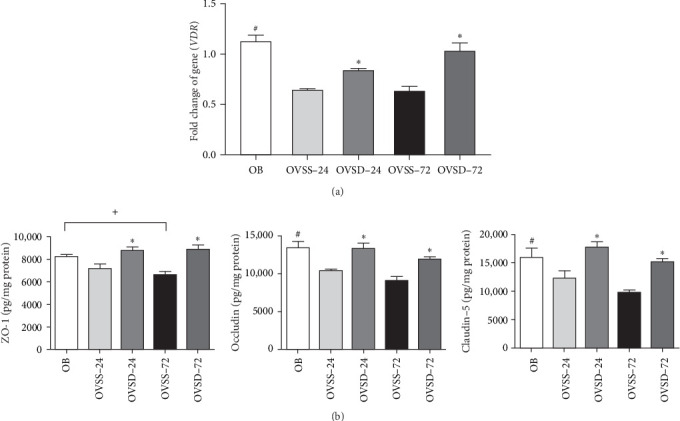
(A) Vitamin D receptor (VDR) expression and (B) tight junction protein levels in the lungs at two time points after cecal ligation and puncture (CLP). Calcitriol treatment enhanced VDR expression and zona occludens (ZOs)-1, occludin, and claudin-5 levels in ovariectomized mice with sepsis. OB, mice with a sham-ovariectomy operation and a high-fat diet (HFD) (*n* = 8); OVSS, mice with an ovariectomy, an HFD, and then CLP was performed, and saline was injected after CLP (*n* = 16), sacrificed on either 24 h (OVSS-24) or 72 h (OVSS-72); and OVSD, mice with an ovariectomy, an HFD, and CLP, and calcitriol was injected after CLP (*n* = 16), sacrificed on either 24 h (OVSD-24) or 72 h (OVSD-72). Values are expressed as the mean ± SEM. Comparisons among groups were analyzed by a two-way ANOVA followed by the Bonferroni post hoc test. ^#^Significantly differs from the OVSS groups on 24 and 72 h. ^+^Significantly differs from the OVSS group on 72 h. *⁣*^*∗*^Significantly differs from the OVSS group at the same time point (*p* < 0.05).

To evaluate the effects of calcitriol on VDR expression and the integrity of the alveolar-capillary barrier, VDR gene expression and some TJs levels were analyzed. We found that an ovariectomy combined with sepsis resulted in lower VDR expression and ZO-1, occludin, and claudin-5 levels. Calcitriol treatment significantly increased VDR expression (Figure 4A) and these three TJ protein levels after CLP (Figure 4B).

### 3.5. Calcitriol Reduces the Iron Content and Improves the GSH Redox Status in Lung Tissues of Ovariectomized Mice With Sepsis

**Figure 5:**
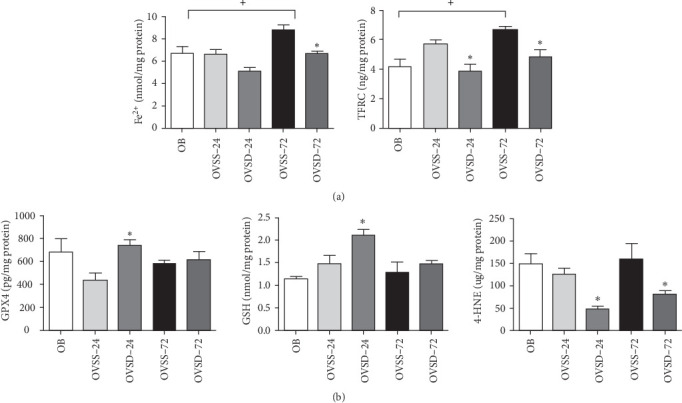
(A) Ferrous iron (Fe^2+^) contents and transferrin receptor (TFRC) levels, (B) glutathione peroxidase 4 (GPX4) activities, reduced glutathione (GSH), and 4-hydroxynonenal (4-HNE) levels in lungs of mice at two time points after cecal ligation and puncture (CLP). Calcitriol treatment reduced Fe^2+^, TFRC, and 4-HNE levels, and enhanced GSH levels and GPX4 activities in ovariectomized mice with sepsis. OB, mice with a sham-ovariectomy operation and a high-fat diet (HFD) (*n* = 8); OVSS, mice with an ovariectomy, an HFD, and then cecal ligation and puncture (CLP) was performed, and saline was injected after CLP, sacrificed on either 24 h (OVSS-24) or 72 h (OVSS-72) (*n* = 8 at each time point); and OVSD, mice with an ovariectomy, an HFD, and CLP, and calcitriol was injected after CLP, sacrificed on either 24 h (OVSD-24) or 72 h (OVSD-72) (*n* = 8 at each time point). Values are expressed as the mean ± SEM. Comparisons among experimental groups were analyzed by a two-way ANOVA followed by the Bonferroni post hoc test. ^+^Significantly differs from the OVSS group on 72 h. *⁣*^*∗*^Significantly differs from the OVSS group at the same time point (*p* < 0.05).

The OVSS group had higher Fe^2+^ and TFRC levels on 72 h post-CLP than did the OB group. Among the groups with an ovariectomy and CLP, the OVSD group had lower Fe^2+^ on 72 h, while TFRC levels were lower on both 24 and 72 h than those in the OVSS groups (Figure 5A). The GPX4, GSH, and 4-HNE levels did not differ between the OB and OVSS groups. However, higher GPX4 and GSH levels on 24 h and lower 4-HNE on both 24 and 72 h post-CLP were noted in the OVSD groups than those in the OVSS groups (Figure 5B)

### 3.6. Calcitriol Attenuates Lung Ferroptosis in Ovariectomized Mice With Sepsis

**Figure 6:**
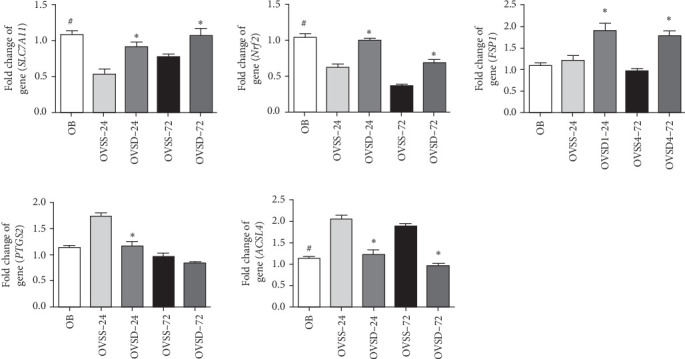
Messenger RNA expressions of ferroptosis-associated components, including solute carrier family 7 member 11 (*SLC7A11*), nuclear factor erythroid 2-related factor 2 (*NRF2*), ferroptosis-suppressing protein 1 (*FSP1*), prostaglandin-endoperoxide synthase 2 (*PTGS2*), and acyl-CoA synthetase long chain family member 4 (*ACSL4*), in lungs of the experimental groups. An ovariectomy concomitant with sepsis significantly increased *ACSL4* and decreased *SLC7A11* and *Nrf2* expression levels. Calcitriol treatment suppressed lung ferroptosis in ovariectomized mice with sepsis. OB, mice with a sham-ovariectomy operation and a high-fat diet (HFD) (*n* = 8); OVSS, mice with an ovariectomy, an HFD, and then cecal ligation and puncture (CLP) was performed, and saline was injected after CLP, sacrificed on either 24 h (OVSS-24) or 72 h (OVSS-72) (*n* = 8 at each time point); and OVSD, mice with an ovariectomy, an HFD, and CLP, and calcitriol was injected after CLP, sacrificed on either 24 h (OVSD-24) or 72 h (OVSD-72) (*n* = 8 at each time point). Values are expressed as the mean ± SEM. Comparisons among experimental groups were analyzed by a two-way ANOVA followed by the Bonferroni post hoc test. ^#^Significantly differs from the OVSS groups on 24 and 72 h. *⁣*^*∗*^Significantly differs from the OVSS group at the same time point (*p* < 0.05).

Mice with an ovariectomy and CLP had significantly lower *SLC7A11* and *Nrf2* and higher *ACSL4* expressions than obese mice with a sham operation. Among groups with an ovariectomy and CLP, calcitriol treatment upregulated *SLC7A11*, *Nrf2*, and *FSP1*, and downregulated *PTGS2* and *ACSL4* expression levels on 24 h or both 24 and 72 h after CLP (Figure 6).

### 3.7. Calcitriol Attenuates Lung Cuproptosis in Ovariectomized Mice With Sepsis

**Figure 7:**
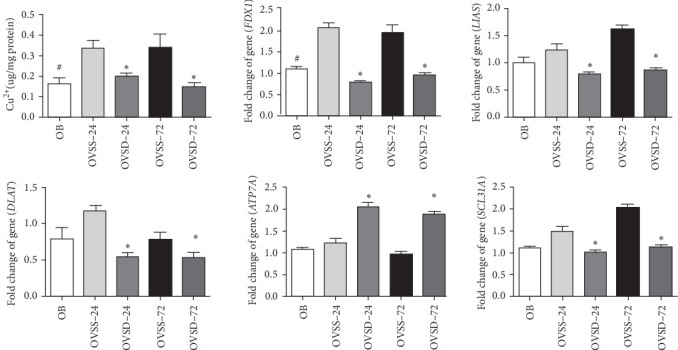
Copper concentrations and messenger RNA expressions of cuproptosis-associated components, including ferredoxin 1 (*FDX1*), lipoic acid synthase (*LIAS*), dihydrolipoyl transacetylase (*DLAT*), ATPase copper transporting alpha (*ATP7A*), and solute carrier family 31 member 1 (*SCL31A*), in lungs of the experimental groups. An ovariectomy concomitant with sepsis significantly increased the copper content and *FDX1* expression. Calcitriol treatment suppressed copper overload and cuproptosis in ovariectomized mice with sepsis. OB, mice with a sham-ovariectomy operation and a high-fat diet (HFD) (*n* = 8); OVSS, mice with an ovariectomy, an HFD, and then cecal ligation and puncture (CLP) was performed, and saline was injected after CLP, sacrificed on either 24 h (OVSS-24) or 72 h (OVSS-72) (*n* = 8 at each time point); and OVSD, mice with an ovariectomy, an HFD, and CLP, and calcitriol was injected after CLP, sacrificed on either 24 h (OVSD-24) or 72 h (OVSD-72) (*n* = 8 at each time point). Values are expressed as the mean ± SEM. Comparisons among experimental groups were analyzed by a two-way ANOVA followed by the Bonferroni post hoc test. ^#^Significantly differs from the OVSS groups on 24 and 72 h. *⁣*^*∗*^Significantly differs from the OVSS group at the same time point (*p* < 0.05).

Cu^2+^ levels and *FDX1* gene expression in the OVSS groups were significantly higher on both 24 and 72 h, and *LIAS* and *SCL31A* expressions were higher on 72 h after CLP than those in the OB group. In groups with an ovariectomy and CLP, mice with calcitriol administration had significantly lower Cu^2+^ contents. Also, *FDX1*, *LIAS*, *DLAT*, and *SCL31A* gene expression levels were lower, while *ATP7A* expression was higher than those expressed in saline-injected groups on 24 h or on both 24 and 72 h after CLP (Figure 7).

### 3.8. Histological Findings of Lung Tissues and Survival Rates

**Figure 8:**
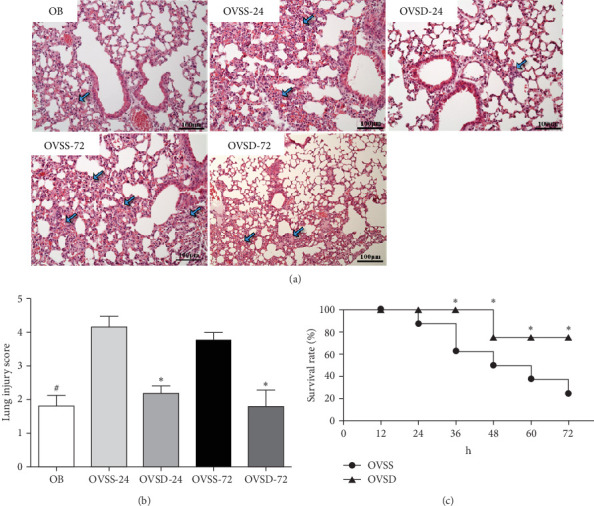
(A) Representative histological images of lung tissues, (B) lung injury scores of the experimental groups, and (C) survival rates of the mice with an ovariectomy and sepsis. An ovariectomy concomitant with sepsis resulted in lung injury, and calcitriol treatment mitigated damage to the lungs and improved survival rates. OB, mice with a sham-ovariectomy operation and a high-fat diet (HFD) (*n* = 8); OVSS, mice with an ovariectomy, an HFD, and then cecal ligation and puncture (CLP) was performed, and saline was injected after CLP, sacrificed on either 24 h (OVSS-24) or 72 h (OVSS-72) (OVSS4) (*n* = 8 at each time point); and OVSD, mice with an ovariectomy, an HFD, and CLP, and calcitriol was injected after CLP, sacrificed on either 24 h (OVSD-24) or 72 h (OVSD-72) (*n* = 8 at each time point). Values are expressed as the mean ± SEM. Comparisons among experimental groups were analyzed by a two-way ANOVA followed by the Bonferroni post hoc test. Survival curves were compared using a log-rank test. ^#^Significantly differs from the OVSS groups on 24 h and 72 h. *⁣*^*∗*^Significantly differs from the OVSS group (*p* < 0.05).

Histological findings of H&E staining showed that lesions and alveolar wall thickening were observed in obese mice, while an ovariectomy concomitant with sepsis aggravated the pulmonary damage. Calcitriol administration alleviated lung injury scores compared to the corresponding group without calcitriol (Figure 8A,B). Survival rates showed that all (100%) of the OVSD group had survived at 24 and 36 h, and 75% at 48 h, which was sustained to 72 h post-CLP. The OVSS group had respective 87.5%, 62.5%, 50%, 37.5%, and 25% survival rates at 24, 36, 48, 60, and 72 h. The OVSD group had significantly higher survival rates than the OVSS group (Figure 8C).

## 4. Discussion

In this experiment, we specifically focused on obese mice concurrence with ovariectomy and sepsis. This emphasis was based on estimations suggesting a high tendency for perimenopausal women to become overweight or obese [3]. A lean control group was not included for comparison, as our previous study had already demonstrated that obese mice exhibit a stronger inflammatory response than their lean counterparts [31]. Other investigations have shown that obese mice are more susceptible to sepsis and experience a higher mortality rate [32, 33]. Furthermore, a clinical study reported that, among sepsis patients, obese individuals had a greater mortality risk than those with normal weight [34]. Given these findings, disease-relevant obese populations warrant focused investigation. In this study, we found that an ovariectomy and sepsis in mice resulted in elevated systemic inflammation, dysregulation of Ang/Tie2 signaling, reduced TJ proteins, and disturbances of the metal ion balance in the lungs. Calcitriol treatment reversed the ovariectomy- and sepsis-induced pulmonary Ang/Tie2 imbalance and alleviated ferroptosis and cuproptosis. Enhanced TJ levels and histological findings also suggested improvements in the alveolar-capillary barrier integrity and lung injury.

Several plasma biomarkers were measured in this study. CXCL10 is a chemokine that is produced during infection and inflammation and is a regulator of lymphocyte activation [35]. MCP-1 and MCP-3 are predominantly localized in the pulmonary epithelium. Both of them are associated with neutrophil activation and trafficking and the pathogenesis of lung injury [36]. IL-22 was identified in various tissues, including the lungs. IL-22 regulates a host's defense at barrier surfaces and plays a role in tissue regeneration [37]. Findings of this study revealed that an ovariectomy with sepsis led to the elevation of these markers, which indicated lung inflammation and epithelial injury. The reduced CXCL10, MCP-1, and MCP-3 and higher IL-22 levels observed in the calcitriol-treated groups suggested attenuation of inflammation and barrier injury in lung tissues.

The pathophysiology of sepsis is a highly complex response that involves activation of various cell types, inflammatory mediators, and associated regulatory systems. The endothelium plays a key role in orchestrating a host's response in sepsis [38]. Vascular endothelium dysfunction is associated with diffuse capillary leakage and the severity of sepsis-induced organ injury [38, 39]. The Ang/Tie2 pathway is one of the important mechanisms correlated with endothelial injury. Angs are proteins with important roles in vascular development and angiogenesis. Ang1 stabilizes the endothelium, suppresses inflammation, and inhibits vascular leakage, while Ang2 has opposing actions to those of Ang1 that disrupt the microvascular integrity, resulting in vascular leakage and end-organ dysfunction [7, 40]. Tie2 is a receptor for Ang proteins that is exclusively expressed by the endothelium. Ang1/Tie2 signaling promotes endothelial cell survival and vascular integrity. In contrast, Ang2 acts as a competitive antagonist of Ang1/Tie2 signaling during inflammation and infections [8, 40]. Previous clinical studies found that elevation of circulating Ang2 is associated with pulmonary vascular leakage and increased mortality in sepsis [41, 42]. The Ang2/Ang1 and Ang1/Tie2 ratios have prognostic significance in identifying clinical outcomes of patients with sepsis [43]. Results of this study showed that Ang1 and Tie2 levels and the Ang1/Tie2 ratio were higher in calcitriol-treated groups. Consistent with these findings, VEGF and TJ protein levels were elevated in groups with calcitriol administration. VEGF is an angiogenic factor that promotes the growth of vascular endothelial cells [44]. Claudin-5, occludin, and ZO-1 are TJ proteins expressed by both epithelial and endothelial cells [45, 46]. Claudin-5 is a membrane protein and a critical component of TJs. Occludin is a transmembrane protein that is localized at bicellular junctions. ZO-1 contains multiple domains that bind to and organize other junctional components in maintaining barrier homeostasis and function. Previous studies found that increases in these TJ protein expressions improved endothelial and epithelial barrier functions [45–48]. These findings suggest that calcitriol administration alleviated pulmonary endothelial injury and improved the integrity of the alveolar-capillary barrier function.

Since ferroptosis and cuproptosis are closely related to sepsis-induced ALI [11, 12], we analyzed several components associated with ferroptosis and cuproptosis to evaluate the impacts of calcitriol on iron and copper homeostasis in lung tissues. Ferroptosis mainly results from iron-dependent phospholipid peroxidation that is related to GSH depletion, GPX4 inhibition, and reactive oxygen species (ROS) accumulation [21]. On the other hand, TFRC promotes iron transport into cells, which facilitates ferroptotic cell death. ACSL4 converts PUFAs into acyl-CoA derivatives, which thereby serve as substrates for lipid peroxidation. PTGS2 is the key enzyme that metabolizes arachidonic acid into prostaglandin, which is upregulated under oxidative stress. TFRC, ACSL4, and PTGS2 are widely used as biomarkers of ferroptosis [49]. FSP-1 is a GSH-independent free-radical scavenger that protects against ferroptosis [50]. SLC7A11, a cystine transporter, which facilitates GSH biosynthesis and antioxidant defense [51]. Nrf2 is a key regulator in maintaining the proper redox homeostasis in cells. Upregulation of Nrf2 prevents lipid peroxidation and ferroptosis [52, 53]. In this study, we found that VD-treated groups had lower Fe^2+^ levels and TFRC, ACSL4, and PTGS2 expressions, whereas protective antioxidative components, including FSP-1, SLC7A11, Nrf2, GSH, and GPX4, were upregulated. These findings suggest that there was a more balanced redox response and ferroptosis was mitigated when calcitriol was administered after CLP.

As to cuproptosis in the lungs, several important regulatory components were evaluated. DLAT is an essential subunit of the pyruvate dehydrogenase complex. Excessive intracellular copper targets and binds to lipoylated DLAT, causing proteotoxic stress and cuproptosis [10]. LIAS is a component of the lipoic acid pathway. FDX1 functions as a reductase to reduce Cu^2+^ to Cu^+^, which directly binds to lipoylated proteins. There is a close correlation among DLAT, LIAS, and FDX1. Gene knockout studies reported that deletion of FDX1 and LIAS leads to a reduction in protein lipoylation and prevents cuproptosis [54]. ATP7A/B and SLC31A1 are copper transporters that regulate intracellular copper homeostasis. ATP7A/B are copper exporters, while SLC31A1 imports copper. One study showed that deletion of *ATP7B* or overexpression of *SLC31A1* resulted in copper accumulation and cell death [21]. The findings of this study revealed that Cu^2+^ concentrations and *FDX1*, *LIAS*, *DLAT*, and *SLC31A1* expression levels in the lungs were reduced, whereas *ATP7A* increased in calcitriol-treated groups. These findings suggest that calcitriol may be involved in mitigating copper overload and alleviating lung cuproptosis induced by an ovariectomy complicated with sepsis.

The mechanisms responsible for the favorable effects of calcitriol on attenuating ALI may partially be derived from the interaction between VD and VDR. Calcitriol is a high-affinity ligand that can directly activate the VDR [55]. Activation of calcitriol/VDR signaling increases TJ protein expressions and improves barrier functions [56]. A previous study showed that VDR deletion resulted in destruction of the pulmonary barrier integrity and increased lung permeability [57]. On the other hand, the VDR was also reported to attenuate ferroptotic changes, thus providing protection against organ injuries [19, 58]. At present, there is no study investigating the effects of VD on cuproptosis. Although our study showed that cuproptosis was suppressed in the calcitriol-treated groups, the findings may result from inhibition of ferroptosis. Ferroptosis and cuproptosis are closely related in signal pathways, and ferroptosis inducers can enhance cuproptosis [21]. The improved redox status and anti-inflammatory effects derived from VD treatment may have contributed to the mitigation of ferroptosis and cuproptosis and improved the integrity of the pulmonary barrier. The histological findings also showed that lung injury was attenuated when calcitriol was administered after sepsis.

There are limitations in this study. First, because CLP-induced sepsis is a well-established model with known clinical implications, an ovariectomy-only group without CLP was not included. On the other hand, the absence of a sham ovariectomy-CLP group limits the ability to delineate the isolated effects of ovariectomy in this sepsis model. As such, the current design reflects the cumulative effects of both ovariectomy and sepsis, rather than attributing outcomes to either condition independently. Second, our investigation centered specifically on the influence of calcitriol treatment in obese mice. The potential differential responses to calcitriol between lean and obese phenotypes under conditions of ovariectomy and sepsis remain unexplored and warrant further investigation.

In conclusion, this study showed for the first time that an ovariectomy concomitant with sepsis resulted in dysregulation of the Ang/Tie2 axis and impairment of the barrier integrity. Also, ferroptosis and cuproptosis were noted in lung tissues. Calcitriol administration elicited a more balanced Ang/Tie signaling and improved the barrier function in the lungs. Alleviated metal ion overload, mitigated ferroptosis and cuproptosis, and enhanced survival were also observed in calcitriol-treated groups when concurrence with ovariectomy and sepsis. This study implies that calcitriol treatment may have potential therapeutic importance in critical conditions in older obese female subjects complicated with ALI.

## Figures and Tables

**Table 1:** Composition of the high-fat diet.

Ingredients (g)	High-fat diet
Casein, lactic	233.06
L-cystine	3.50
Corn starch	84.83
Maltodextrin	116.53
Sucrose	206.02
Cellulose	58.26
Lard	206.84
Soybean oil	29.13
Mineral mix^a^	58.26
Choline bitartrate	2.33
Vitamin mix^b^	1.17
Dye	0.06
Total	1000
Protein/fat/carbohydrate (kcal %)	20/45/35
Energy density (kcal/g)	4.7

^a^The composition of mineral mixture is listed as follows (g/1000 g): potassium citrate, 330; calcium phosphate, 260; calcium carbonate, 110; sodium chloride, 51.8; magnesium sulfate, 51.52; magnesium oxide, 8.38; ferric citrate 4.2; manganese carbohydrate hydrate, 2.45; zinc carbonate, 1.12; chromium potassium sulfate, 0.39; copper carbonate, 0.21; ammonium molybdate tetrahydrate, 0.06; sodium fluoride, 0.04; sodium selenite, 0.01; potassium iodate, 0.01.

^b^The composition of vitamin mixture is listed as follows (g/100 g): vitamin E acetate, 10; niacin, 3; biotin (1%), 2; pantothenic acid, 1.6; vitamin D3, 1; vitamin B12, 1; vitamin A acetate, 0.8; pyridoxine HCL, 0.7; riboflavin, 0.6; thiamine HCL, 0.6; folic acid, 0.2; menadione sodium bisulfite, 0.08.

**Table 2:** Sequences of the primers used for PCR amplification in the experiment.

Gene name	Primer sequence (5′→3′)		Accession number
*ACSL4*	F:	CCACACTTATGGCCGCTGTT	NM_019477.3
	R:	GGGCGTCATAGCCTTTCTTG	
*ATP7A*	F:	TGGGAAAGTGAATGGTGTCCA	NM_001109757.2
	R:	ACGGTATTGGTTAAGACAGGGA	
*DLAT*	F:	TCACAGACATCCCCATCAGCA	NM_145614.4
	R:	TTAAGTTCCTTCCGTACCAACAG	
*FDX1*	F:	ACAGACAGGAACCTGGAAGACC	NM_001301728.1
	R:	GAGACAATCTGTATGGGGTGGTT	
*FSP1*	F:	CCAGGTGGAAGGTTACAGCA	NM_153779.2
	R:	GGTTGGTCAGTCTCTGGCTTG	
*LIAS*	F:	CGTTAAGACCGCAAGAAATCC	NM_001310612.1
	R:	CCACATCATCTCGATCCACC	
*Nrf2*	F:	CAGCATAGAGCAGGACATGGAG	NM_010902.5
	R:	GAACAGCGGTAGTATCAGCCAG	
*PTGS2*	F:	TGCTGGTGGAAAAACCTCGT	NM_011198.5
	R:	AAAACCCACTTCGCCTCCAA	
*SLC31A1*	F:	GCCTTCGTGGCAGTGTTTTTA	NM_175090.4
	R:	GCGAATGCTGACTTGAGACTTTC	
*SLC7A11*	F:	AAATACGGAGCCTTCCACGA	NM_011990.2
	R:	CTCCAGGGGCAGTCAGTTAG	
*VDR*	F:	ACCAGCTCTACGCCAAGATG	NM_009504.4
	R:	CTTCATGCTGTTCTCCGGCT	
*GAPDH*	F:	AACGACCCCTTCATTGAC	M32599.1
	R:	TCCACGACATACTCAGCAC	

Abbreviations: *ACSL4*, acyl-CoA synthase long chain family member 4; *ATP7A*, ATPase copper transporting alpha; *DLAT*, dihydrolipoyl transacetylase; *FDX1*, ferredoxin1; *FSP1*, ferroptosis-suppressing protein 1; *GAPDH*, glyceraldehyde 3-phosphate dehydrogenase; *LIAS*, lipoic acid synthase; *Nrf2*, nuclear factor erythroid 2-related factor 2; *PTGS2*, prostaglandin-endoperoxide synthase 2; *SLC31A1*, solute carrier family 31 member 1; *SLC7A11*, solute carrier family 7 member 11; *VDR*, vitamin D receptor.

## Data Availability

The data used to support the findings of this study are available from the corresponding author upon request.
